# Tonic Neuromuscular Processing Affects Postural Adaptation Differently in Aging and Parkinson's Disease

**DOI:** 10.3389/fneur.2018.01130

**Published:** 2019-01-21

**Authors:** W. Geoffrey Wright

**Affiliations:** Neuromotor Sciences Program, College of Public Health, Temple University, Philadelphia, PA, United States

**Keywords:** postural tone, rigidity, Parkinsion's disease, basal ganglia, pedunculopontine tegmental nucleus, dopamine

## Abstract

The combination of phasic and tonic neuromuscular processes are involved in the maintenance of normal upright posture. The latter is of particular importance in some pathologies, such as Parkinson's Disease (PD), which is known by one of its cardinal symptoms—tonic dysfunction (i.e., rigidity). Changes in tonic function may also occur during healthy aging. In this investigation, somatosensory input was manipulated by changing the support surface orientation for prolonged periods of quiet stance (QS). The aim was to shed light on how long-term tonic responses called postural lean after-effects are affected by aging and age-related neuropathology. Forty one participants were tested: 19 healthy young (25±5 years), 13 healthy older (63±8 years), and 9 adults with PD (63±5 years). Baseline conditions were eyes-closed QS on a stable surface or standing on an unstable, sway-referenced (SR) surface. Four experimental conditions combined two types of toes-up ramp tilt adaptation (120 s of toes-up static 7° tilt or sinusoidal 7° ± 3° tilt) with two types of post-adaptation (120 s of QS or SR). Results revealed postural after-effects during post-adaptation QS showing significant anterior COP shift for both young and older adults (*p* < 0.0001), but not PD (p > 0.06, n.s.). Compared to young, postural after-effects in older adults showed longer decay constants and did not return to baseline COP within the 120 s post-adaptation period (*p* < 0.05). Postural after-effects during SR, which appeared as toes-up surface tilt were highly significant in healthy populations (*p* = 0.001), but took longer to develop in PD. Younger adults showed significantly larger dorsiflexion (*p* < 0.01) and faster decay constants than older adults (*p* < 0.05). In summary, (1) postural after-effects decayed to baseline when post-tilt surface was stable but were retained and even grew larger post-adaptation in the SR surface conditions in all groups, (2) postural after-effects differed between healthy age groups, (3) PD showed less adaptation to surface changes. Differences in size and decay of after-effects between healthy and PD groups suggest tonic neuromuscular processes play a role in how adaptable postural control is to changing surface conditions and this is affected by healthy aging and basal ganglia function.

## Introduction

Normal upright posture relies on accurately determining the orientation of the support surface with respect to gravity (1–3). While it is well-understood that phasic processes reliant on short latency automatic and reflexive pathways are important to posture and gait, background tonic neuromuscular activity also plays an important role in postural control and locomotion (4–7). Sustained fatigue-resistant muscle activity, referred to as tonus and more specifically postural tone, counteracts gravity and keeps the numerous body segments appropriately aligned in order to maintain upright, stable posture (8). The importance of tonic drive for healthy motor functioning has long been recognized in clinical practice, where tests of muscle tone are considered a highly sensitive sign for measuring central nervous system health. In many, neuropathologies, such as stroke, multiple sclerosis, and Parkinson's Disease (PD), muscle tone is used to assess health status and guide treatment (9–11). In fact, a cardinal symptom of PD is tonic dysfunction, which manifests as rigidity or hypertonicity (12, 13). This type of tonic dysfunction contributes to disabilities affecting balance, locomotion, and increased fall-risk (14–16). Changes in muscle tone and to the processes that control tonic level are present even in healthy aging, which can increase fall risk (17, 18). Falls in the elderly, whether healthy or neurologically impaired, are a leading cause of injury-related death and non-fatal hospitalization, with direct costs related to falls in older adults exceeding $30 billion per year in the US (19).

Postural control has been described as a sustained contraction produced by a descending tonic drive from tonigenic sub-cortical structures (20). A number of brainstem regions are thought to be involved in these tonic neuromuscular processes, which include mesopontine regions that have connectivity to the spinal cord, the basal ganglia, cerebellum, and cortical sensorimotor areas such as M1 and the supplementary motor area (7, 21). Basal ganglia-brainstem-spinal pathways have been identified in the regulation of postural tone and locomotion (6, 7, 22). Specifically, pathways from the substantia nigra pars reticulata (SNpr) to the pedunculopontine tegmental nucleus (PPTN) play an important role in regulating postural tone (7). The mesencephalic locomotor region (MLR) is also closely linked to SNpr and is important for preparing the postural system to begin locomotion (7). While much of this work was performed on animal models, there is also evidence from human case studies and surgical intervention. For example, a lesion in the posterolateral mesopontine tegmentum can also affect tone; the typical decrease in muscle tone (i.e., atonia) during REM sleep does not occur following damage to this region (23). A lesion to the more medial part of the pedunculopontine complex impairs the ability to stand and walk (24). The PPTN has also been used as a site for deep brain stimulation (DBS) surgery in PD patients (25, 26), which has shown positive impact on postural tone and gait (27). Identifying these tonogenic structures has shed some light on their involvement in postural control, but the importance of these subcortical processes is under-appreciated.

Systematically studying how tonic drive influences postural control in humans can be challenging because mechanical perturbations to the system must be long-lasting and behavioral measures must be sensitive enough to differentiate among short-term phasic responses (e.g., stretch reflexes), volitional interference due to conscious awareness of change, and the long-term tonic adaptations that one is interested in. One means of investigating this has been accomplished by examining the muscle set-points. A muscle set-point can be thought of as the postural tone of flexor and extensor muscle activity about a joint used to maintain a body part in a position, e.g., postural maintenance. While control of muscle set-points can show segmental autonomy, it is thought that in an intact system this is ultimately under the control of central command (8). Changes in set-point can be seen in lean after-effects, which have provided a useful behavioral technique for investigating tonic postural control. A lean after-effect is seen when normal upright stance, which typically aligns with gravity and is orthogonal to a horizontal surface, is altered (3, 28). Use of the support surface as a somatosensory reference for orientation is seen by alignment of the body to the surface when a surface is slowly tilted (2, 29, 30). However, this reference changes if it is altered for an extended period of time, for example, by standing on a stable, tilted surface or a dynamically tilted surface for a few minutes. The surface-to-body angular relation after a surface is tilted toes-up will be maintained in a dorsiflexed position when the surface is returned to horizontal, thus resulting in forward lean. This after-effect can also be observed on a sway-referenced (SR) surface, whereby the individual adopts a surface tilts toes up posture during the post-adaptation period (3). These lean after-effects and surface-tilt after-effects together fall under the umbrella term of postural after-effects. In both cases, they presumably represent a recalibration in postural reference frame with a new tonic set-point of muscle activity. Evidence that this after-effect is centrally driven is suggested by the fact that global postural variables, not simply local muscle group set-point, are altered (31). If centrally driven, this raises the question of how central disease or pathology might affect changes in set-point.

The goal of this study is to determine if tonic processes underlying postural control change across the lifespan or with disease by investigating postural after-effects in healthy young adults, healthy older adults, and adults with PD. The presence, magnitude, and time course of postural after-effects are assumed to be an indicator of some of the variables that drive tonic neuromuscular processing. Specifically, postural after-effects represent an ability to adapt to long-lasting sensory inputs, i.e., tonic input, which help define a set-point from which phasic activity originates. In other words, the starting point for a phasic movement is defined by an “initial condition” which is set by tonic control. The importance of investigating changes in postural adaptation is that dysfunction in tonic control likely plays a significant role in motor, postural, and gait deficits, which both healthy older (32) and to a greater degree PD populations must contend (15, 16, 33). As previous studies have shown, it may not simply be the presence of hypertonicity that causes postural instability and gait dyscoordination (34, 35), but rather how adaptable the motor system is when changing from one state to another (36). Greater understanding of these relationships could, in turn, lead to new and innovative treatment approaches that improve the standard of living for our ever-growing aging population.

## Materials and Methods

### Participants

Nineteen healthy, young participants (20–32 years old, 9 M), 13 healthy older adults (50–74 years old, 8 M), who were age and gender-matched to the PD participants, and 9 individuals with PD (53–70 years old, 6 M) participated in this study. All healthy participants had no known neuromuscular impairment, and no history of PD, neurological disease, or sensorimotor deficits. All PD participants were classified as Hoehn and Yahr 2–3 and were responsive to anti-Parkinson drug treatment as verified by the referring neurologists. Parkinson's Disease (PD) participants were tested ON medication. Participants were included only if able to stand unassisted for at least 10 min periods of time and had normal ankle range of motion (Dorsiflexion = 12°, Plantarflexion = 55°). The protocol was approved by the local IRB at Temple University, and all participants gave written, informed consent before participating in this study. Investigators adhered to the policies regarding protection of human participants as prescribed by the Helsinki Accords.

### Protocols

All data was collected using a 3-degree of freedom (DOF) posture platform (Neurocom Inc.) with integrated dual triaxial AMTI (Watertown, MA) force plates. All conditions required participants to stand in a relaxed stable, upright posture with arms hanging comfortably to the sides with eyes-closed. In the traditional quiet standing (QS) conditions, the surface was fixed in place to provide a stable support surface during which center-of-pressure (COP) time series data was collected. During surface sway-referencing (SR) trials, the servo-driven surface has a pre-programmed capability to allow the surface to tilt with reference to changes in the COP. To ensure proper function of the surface SR, the participant's lateral malleoli were aligned with the rotation axis of the tiltable surface at the center of the force plate. Thus, the zero point for COP was aligned with the participant's ankle with positive COP values representing points anterior to the rotation/ankle axis.

**Figure 1:**
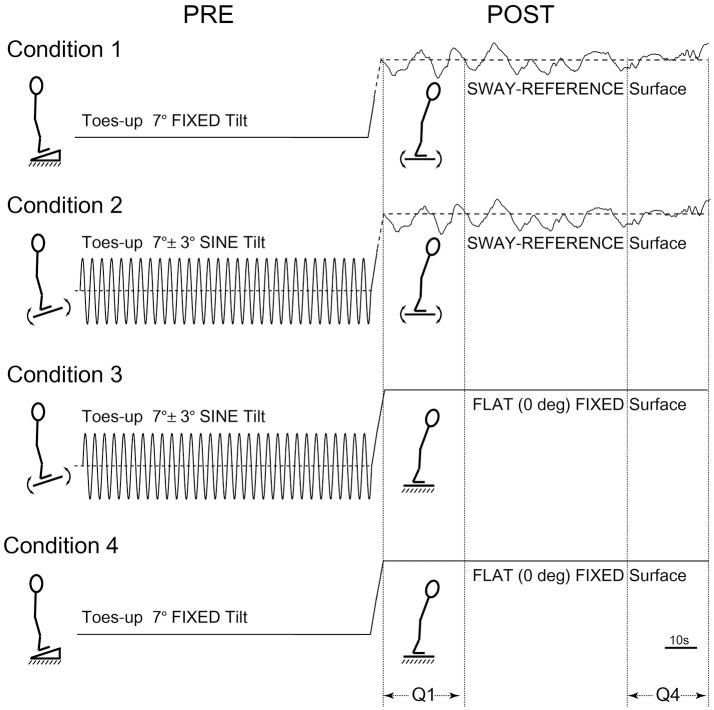
Postural test conditions. Condition 1: toes-up static-ramp for 120 s followed by standing on a sway-referenced surface for 120 s. Condition 2: toes-up sine-ramp (7° ± 3° * $\sin\frac{\pi t}{2}$ at 0.25 Hz) for 120 s followed by standing on a sway-referenced surface for 120 s. Condition 3: toes-up sine-ramp (7° ± 3° * $\sin\frac{\pi t}{2}$ at 0.25 Hz) for 120 s followed by quiet-stance on a static flat surface for 120 s. Condition 4: toes-up static-ramp (7°) for 120 s followed by quiet-stance on a static flat surface (120 s).

The order of tests for participants first involved baseline testing in two 120 s conditions: Eyes-closed on a firm surface, and eyes-closed on a SR surface, i.e., forward sway causes the surface to tilt toes-down in an amount proportional to the forward COP shift, and vice versa. Participants were then tested in four conditions each lasting 240 s. The four counter-balanced conditions were as follows: (1) toes-up static-ramp for 120 s followed by standing on a SR surface for 120 s, (2) toes-up sine-ramp (7° ± 3° ^*^ $\sin\frac{\pi t}{2}$ at 0.25 Hz) for 120 s followed by standing on a SR surface for 120 s, (3) toes-up sine-ramp (7° ± 3° ^*^ $\sin\frac{\pi t}{2}$ at 0.25 Hz) for 120 s followed by quiet-stance on a static flat surface for 120 s, (4) toes-up static-ramp (7°) for 120 s followed by quiet-stance on a static flat surface for 120 s (Figure 1). All conditions were tested eyes-closed, shoes and socks removed, and a harness secured to the ceiling was used to prevent falls without restricting movement.

### Data Collection and Analysis

Time series data was collected at 200 Hz sampling rate for all dependent variables (DV). In the static, flat surface conditions, only COP (cm) was collected since surface tilt equals zero at all times. In SR trials, COP and surface tilt (degrees) were collected. Only anterior-posterior (AP) COP was analyzed since all perturbations were along the AP axis. AP COP was measured relative to the axis of surface rotation. During SR trials, the surface is driven in response to the movement of the participant's COP with a gain of one by converting COP linear movement into a surface tilt angle normalized relative to the participant's height. Therefore, the COP data in the AP direction closely matches the surface orientation data in degrees, however, the zero-point of the surface orientation is able to change with a net shift in center of mass, while the phasic patterns of the COP and surface orientation remain similar with minimal low-pass filtering and negligible phase lag. Because data for all subjects in all four conditions were not available, either due to subject fatigue (in PD group), equipment failure, or data loss, the data from matching post-adaptation conditions (SR: Cond 1 pooled with Cond 2; Static flat surface: Cond 3 pooled with Cond 4) were pooled after it was determined that they did not differ significantly (*p* > 0.34, n.s.). All subsequent analyses were performed on pooled data.

Postural after-effects were compared using a 3 × 3 (time-by-group) mixed model repeated-measures analysis of variance (rmANOVA). The three time periods were baseline (an average of first and last quartile of the baseline time series), first quartile (Q1) of the 120 s period after the ramp adaptation, and last quartile (Q4) of the 120 s period after the ramp adaptation (3). When significant differences were found in the 3 × 3 rmANOVA, subsequent 2 × 3 and univariate rmANOVA and planned comparisons were tested to determine where these differences were. Specifically, following prolonged stance on a toes-up tilted surface during the adaptation phase (i.e., the first 120 s), the surface was returned to its flat position and the time series data was measured during the post-adaptation phase. This second 120 s was divided into four quartiles. The amplitude of lean after-effects was analyzed by comparing the average AP COP position between baseline and Q1 using a 2 × 3 rmANOVA. The decay of lean after-effects was analyzed by comparing the average AP COP position at Q1–Q4 using a 2 × 3 rmANOVA. To determine if the postural after-effect completely decayed after 120 s, Q4 was compared to baseline. In the SR conditions, the analysis of the amplitude and decay of surface-tilt after-effects was performed in a similar manner, the only difference being that average surface tilt angle at baseline, Q1, and Q4 was used. Significance was set at *p* ≤ 0.05.

## Results

### Baseline

Baseline AP COP was significantly different across groups [*F*_(2,37)_ = 4.92, *p* = 0.013, η^2^ = 0.21]. PD participants showed a significantly greater anterior position of the AP COP (7.27 ± 0.59 cm), relative to healthy age-matched controls (5.79 ± 0.46 cm). The young adult group showed the smallest anterior distance between the center of the force plate and AP COP (5.06 ± 0.38 cm), but was not different from the healthy older baseline COP (*p* = 0.23, n.s.). The average surface tilt during baseline SR measures showed no difference between groups [*F*_(2,37)_ = 0.93, *p* = 0.40, n.s.).

### After-Effects on a Fixed Surface

**Figure 2:**
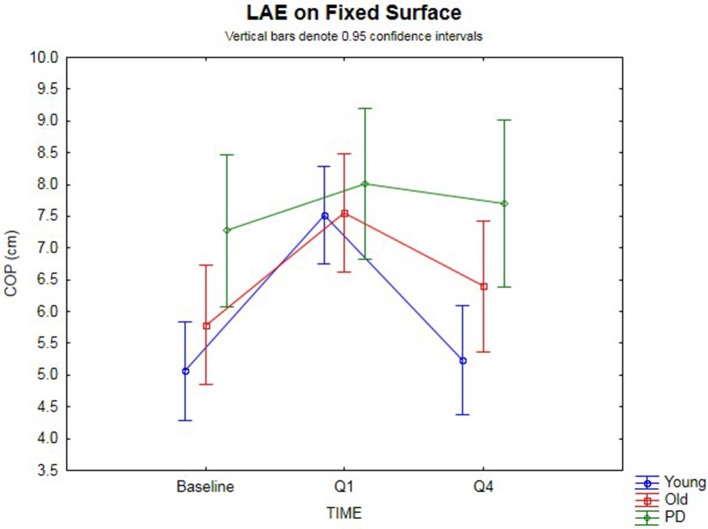
Fixed surface COP during QS. Lean after-effects as measured by the AP COP position on a fixed surface at baseline, then following ramp tilt adaptation at Q1 and Q4. Q1 represents the first 30 s of post-tilt adaptation, which starts immediately after the 120 s of ramp tilt adaptation. Q4 is the last 30 s of the post-tilt adaptation period. The three lines represent each group (Young, Old, PD ON-meds). A positive value represents a forward shift in COP. The post-tilt adaptation values are the average of two trials—QS following fixed ramp tilt adaptation, QS following sine ramp tilt adaptation.

The 3 × 3 rmANOVA showed significant main effects [*F*_(2,74)_ = 24.4, *p* < 0.00001, η^2^ = 0.40] and a significant time-by-group interaction [*F*_(4,74)_ = 3.35, *p* = 0.014, η^2^ = 0.15] (Figure 2). Looking specifically at the AP COP shift between baseline and Q1 (i.e., lean after-effect) while standing on a fixed surface after the 120 s of ramp tilt adaptation, a significant effect was found [*F*_(1,37)_ = 47.4, *p* < 0.00001, η^2^ = 0.56]. A significant time-by-group interaction was found [*F*_(2,37)_ = 4.19, *p* = 0.023, η^2^ = 0.18]. Planned comparisons revealed the young (*p* < 0.00001) and old (*p* < 0.0003) groups both had significant lean after-effects. While the PD group did not show a significant difference using an analysis of variance [*F*_(1,7)_ = 5.02, *p* = 0.06, n.s.], 7 out of the 8 PD participants (Exact binomial, *p* = 0.031) showed a forward shift in the AP COP, which is in the same direction as the healthy cohorts. The amplitude of lean after-effects of the older adult group were larger on average than the in PD group, but did not reach significance [*F*_(1, 19)_ = 4.02, *p* = 0.059, η^2^ = 0.17, n.s.]. However, the healthy young adult group showed a significantly larger lean after-effect than the PD group [*F*_(1,25)_ = 7.38, *p* = 0.012, η^2^ = 0.23]. No difference was found between the younger and older healthy adult groups [*F*_(1,30)_ = 1.64, *p* > 0.10, n.s.].

The decay of the lean after-effect when standing on a fixed surface was analyzed by comparing AP COP at Q1 vs. Q4 (Figure 2). This revealed that the after-effects significantly decayed back toward the baseline over the course of 120 s post-adaptation period [*F*_(1, 37)_ = 33.8, *p* < 0.00001, η^2^ = 0.48]. Planned comparisons by group revealed the healthy younger (*p* < 0.00001) and older (*p* = 0.0024) adult groups both showed significant decay back toward baseline. However, the PD group did not decay during the post-adaptation period (*p* = 0.50, n.s.), despite having shown a small, albeit non-significant lean after-effect. These group differences were further substantiated by a significant time-by-group interaction [*F*_(2, 37)_ = 7.54, *p* < 0.002, η^2^ = 0.29]. When comparing the change from Q1 to Q4, the PD group did not change significantly, but the older healthy adult group did when compared to the PD group [*F*_(1, 19)_ = 4.77, *p* = 0.042, η^2^ = 0.20], and the younger group decayed even more than the older group [*F*_(1, 30)_ = 5.25, *p* = 0.029, η^2^ = 0.15], all suggesting differences in adaptation. There was no difference between baseline and Q4 for all groups [*F*_(1, 37)_ = 2.07, *p* = 0.15, n.s.] suggesting a return to baseline COP had occurred.

### After-Effects on a Sway-referenced Surface

**Figure 3:**
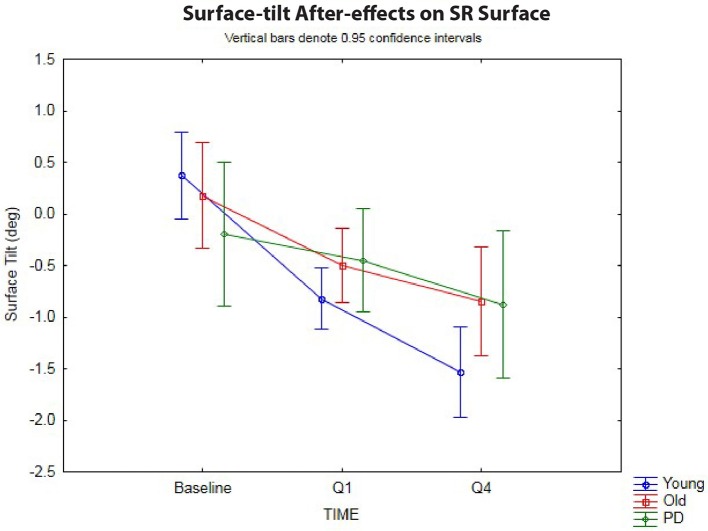
Sway-reference surface tilt. Postural after-effects as measured by the degree of average surface tilt on a sway-referenced surface at baseline, then following ramp tilt adaptation at Q1 and Q4. Q1 represents the first 30 s of post-tilt adaptation, which starts immediately after the 120 s of ramp tilt adaptation. Q4 is the last 30 s of the post-tilt adaptation period. The three lines represent each group (Young, Old, PD ON-meds). A negative value represents a toes-up (dorsiflexed) surface tilt. The post-tilt adaptation values are the average of two trials—SR following fixed ramp tilt adaptation, SR following sine ramp tilt adaptation.

The 3 × 3 rmANOVA showed significant main effects across time [*F*_(2,72)_ = 37.4, *p* < 0.00001, η^2^ = 0.51] and a significant time-by-group interaction [*F*_(4,72)_ = 4.27, *p* < 0.004, η^2^ = 0.19] (Figure 3). Looking specifically at the initial surface-tilt after-effect in Q1, a significant toes-up change in surface-tilt between baseline and Q1 was observed while standing on a SR surface after the 120 s of ramp tilt adaptation [*F*_(1,36)_ = 21.8, *p* = 0.00004, η^2^ = 0.38]. Planned comparisons revealed that the healthy younger (*p* < 0.00001) and older (*p* = 0.008) adult groups both had significant postural after-effects in Q1 relative to baseline, but the PD group did not (*p* = 0.44, n.s.). The size of these after-effects were significantly different as revealed by a time-by-group interaction [*F*_(2,36)_ = 3.37, *p* = 0.046, η^2^ = 0.16]. Specifically, the tilt surface amplitude in the older adult group did not differ significantly from the younger adult [*F*_(1,30)_ = 2.62, *p* = 0.12, n.s.] or PD [*F*_(1,18)_ = 1.58, *p* = 0.23, n.s.] groups at Q1 during the post-adaptation period, but the younger adult group showed a significantly larger toes-up surface tilt than the PD group [*F*_(1,24)_ = 4.98, *p* = 0.035, η^2^ = 0.17].

The decay of the postural after-effects when standing on a SR surface was analyzed by comparing the angle of SR surface tilt at Q1 vs. Q4 (Figure 3). Unlike in the fixed surface post-adaptation conditions, the after-effects in the SR conditions grew significantly over the course of 120 s post-adaptation period [*F*_(1,36)_ = 47.1, *p* < 0.00001, η^2^ = 0.57]. In other words, the surface tilt after-effects did not decay during post-adaptation period, but instead showed a significant increase away from baseline, as has been shown before in healthy young adults (3). Planned comparisons revealed that all three groups, young (*p* < 0.00001), old (*p* < 0.0005), and PD (*p* < 0.01) showed a significant increase in toes-up surface tilt from Q1 to Q4 during 120 s post-adaptation period, however, the size of these after-effects were different across groups as revealed by the significant group-by-time interactions [*F*_(2,36)_ = 3.27, *p* < 0.05, η^2^ = 0.15]. Planned comparisons revealed that young adults showed a larger increase in toes-up surface tilt from Q1 to Q4 relative to the older adults [*F*_(1,30)_ = 5.24, *p* = 0.029, η^2^ = 0.15], but there was no difference between the older adult and PD groups for this variable [*F*_(1,18)_ = 0.22, *p* = 0.64, n.s.].

## Discussion

Differences in postural adaptation were found between young and old age groups and between healthy and PD groups. These findings provide evidence that changes in postural adaptation exist not only when changes in tonic neuromuscular processing due to PD are present, but also due to healthy aging. The tonic differences were seen in both the initial adaptation resulting in lean after-effects, and in the decay pattern following the removal of the tonic tilt input. Significant postural after-effects were seen in all conditions for the healthy groups, regardless of the ramp adaptation (fixed or sine ramp) or the post-adaptation condition (fixed or sway-referenced surface). However, when comparing the healthy older adults to the younger adults, the older adults showed a significantly different decay in their after-effects. When looking at the PD results, this group showed only a small, non-significant change in AP COP on average, and when tested on the SR surface, they also did not show a significant increase in surface tilt between the baseline and the 1st quartile, as was observed in the healthy groups. The only significant postural after-effect in the PD group was an increase in surface tilt between the 1st and 4th quartiles. Together these PD group results suggest that the tonic adaptability is not completely absent but it occurs to a much lesser degree in PD. These findings are discussed further in the following sections.

### Postural After-Effects in Healthy Adults Affected by Age

Postural after-effects were present in all the healthy participants, which far exceeds the 50% prevalence reported when only a fixed ramp adaptation and fixed flat surface post-adaptation was employed in previous studies (28); their approach only allowed for lean after-effects, but did not investigate surface-tilt after-effects. By employing the techniques described in an earlier study (3), it was established that postural after-effects can be induced in most individuals. Using an experimental technique able to induce postural after-effects with such high prevalence was important when comparing between populations, since this reduced the need for extremely large sample sizes, and decreased the risk of being under-powered and missing effects (Type 2 errors). The use of a SR surface during the post-adaptation to measure postural after-effects addresses some of the limitations of the normal test of lean after-effects that had been performed on a fixed surface. For a lean after-effect to appear on a fixed surface, this requires that the test participant moves out of alignment with the gravitational vertical, e.g., forward lean. Maintenance of normal upright posture is accomplished using sensory input from vision, vestibular, and somatosensory inputs. In an eyes-closed paradigm, an individual might detect that they are leaning, rather than standing straight upright, using proprioceptive input from the feet soles and ankles and graviceptive inputs from the otoliths and specific internal organs (1, 37). Therefore, an individual may resist the lean after-effect because they are aware of being misaligned with vertical or even sense that they are approaching their limits of stability and are at risk of falling. Unconsciously, automatic postural processes may also be responsible for tonic changes in the postural set-point, causing the lean after-effect to dissipate quickly or not appear at all. When using a SR surface during the post-adaptation period, muscle set-points in the various body segments can be adopted without significantly moving the center-of-mass toward the limits of stability. This may account for the much higher prevalence of postural after-effects in the current populations.

Despite the fact that both the healthy older and younger adults all showed postural after-effects, when the amplitude and temporal dynamics of this adaptation process was analyzed, differences between age groups were found. At baseline, there were no significant differences in AP COP position or surface tilt (*p*>0.10) that could account for a postural predisposition toward leaning. The younger adult group showed a faster return toward baseline COP in the fixed surface post-adaptation condition than the healthy older adults. This suggests that despite having a larger after-effect, they were able to efficiently use the reliable vestibular and somatosensory inputs to recalibrate their postural vertical relative to the fixed horizontal surface during the post-adaptation period. There was also a persistence of the surface-tilt after-effect on the sway-reference surface during post-adaptation period in younger adults that was greater than in the healthy older adults. This persistent and increasing postural after-effect can be seen as adaptive because the SR surface is not a reliable reference for maintaining upright posture and the vestibular vertical stays aligned with gravity throughout the postural after-effect in the SR condition. Therefore, the postural system maintains its last reliable set-point, i.e., dorsiflexed ankle. While the underlying physiological mechanism for this difference was not examined in the age-dissociated populations, one possible contribution to this difference in tonic behavior could be due to age-related loss of dopamine in healthy individuals. Even in older adults without PD, dopamine loss occurs at a rate of 7% per decade (38–40). This loss has been correlated with motor and postural control impairments (17, 41). Imaging studies suggest that this decrease in dopamine is related to the loss of dorsal SN cells in healthy older adults (42). The current evidence may provide further evidence of a behavioral link between the dopaminergic system, tonic neuromuscular processes, and changes in postural and motor control in a healthy aging population. Furthermore, when dopamine loss reaches pathological levels such as in PD, the effects of these tonic differences become even more pronounced, as discussed below.

### Postural-After Effects in Parkinson's Disease

In addition to the evidence that aging alters postural adaptation, this study suggests basal ganglia disease does as well. The size and decay of postural after-effects in adults with PD were significantly different from healthy adults. Specifically, the size of after-effects was smaller than the healthy older and younger adults. And unlike in the healthy adults who showed decay of lean after-effects back to baseline when standing on the fixed surface, the small lean after-effects that the PD group showed did not decay back to baseline within the 120 s post-adaptation period. A similar finding on the SR surface occurred in that the PD's small toes-up surface-tilt after-effects did not change during the post-adaptation period. This suggests that PD's show less postural adaptation and it takes longer to change the posturally-relevant tonic muscle set-point than in healthy adults.

Difficulty in regulating appropriate levels of background postural tone has been shown before by using fast, external perturbations (36), but the current study shows that this difficulty extends far beyond phasic response time-scales. The conditions tested here were on the order of minutes and while healthy adults were able to adapt to new tonic neuromuscular inputs within tens of seconds, the PD group in some cases were unable to adapt for periods 10 times longer than that. Although, it is well-understood that phasic processes reliant on short latency, reflexive pathways and late occurring automatic postural responses affect posture and gait in PD (43, 44), the current study provides new evidence that tonic neuromuscular processing also plays an important role in postural adaptation. The background muscle tone present during postural maintenance is thought to provide a sustained muscle activity needed to counteract gravity and keep the numerous body segments appropriately aligned (1). The current study adds to the growing body of evidence that deficits in postural tonic control, especially in the axial musculature (15, 16) can play an important role in balance deficits in PD.

The dopamine system's role in postural tone and motor control in healthy and PD populations is known (7, 36, 44) and there is evidence that dopamine treatment can help posture and gait control in PD (45). However, there are numerous studies showing that these behaviors can be resistant to dopamine therapy even though other symptoms abate (44, 46). The origin of such prolonged tonic muscle contraction is thought to come from sub-cortical and brainstem structures (20), which are tightly connected to nigrostriatal regions. Among these structures are mesopontine tegmental regions with descending and ascending connectivity (21). The involvement of these neural regions in posture and gait has led researchers to use the PPTN as a site for implanting DBS electrodes in PD patients (25, 26). Although this has had mixed success [(47, 48)], at least a few studies have shown reduction in axial tone and improvement in the symptoms of Postural Instability and Gait Disturbance (PIGD) (27). While results from the current study provide some additional insight into how changes in function of the basal ganglia and associated nuclei can affect postural behavior, its likely that tonic dysfunction observed in PD is the result of a widely distributed cortical/subcortical network (49). Further evidence is needed to determine how effective targeted treatment of only the dopamine system may be, since standard dopaminergic pharmacological treatment of PD has in some cases been shown to have little effect on axial tone or symptoms related to posture and gait (15, 16, 44, 46). Studies are underway, which involve testing PD both ON and OFF medication in the current experimental procedure, which may provide additional insight into this question.

A number of alternative explanations were considered, all of which could not be completely ruled out. These findings presuppose that all participants had no limitation in their ankle joint, which was verified by screening all participants for normal ankle ROM. All participants were able to maintain dorsiflexion by standing un-aided on the toes-up tilted surface for the full 120 s adaptation period. In the sine-ramp tilted condition, this required at least 10° dorsiflexion, which is almost an order of magnitude greater than the size of the after-effect. Another explanation that can be ruled out is the forward lean that the PD showed at baseline. A symptom of PD is excessive kyphosis, marked by a forward stooped-posture, and there is some evidence (50), albeit mixed (51), that this inherent anterior flexion contributes to deficits in automatic postural stabilization. The presence of a significant anterior baseline shift in the PD group may have limited how large the lean after-effect could shift forward before reaching the limits of stability, however, the decay timeline still differed from the healthy group. Furthermore, during the post-adaptation period, the lean after-effect failed to return as quickly as the healthy adults. Additionally, in the baseline SR condition, there was not a difference between groups. Another factor that could contribute to group differences in the after-effects is the presence of proprioceptive deficits in PD. These proprioceptive deficits may affect the ability to correctly orient stance relative to vertical and/or the surface (52–54). They may also be reflective of more general sensorimotor integration problem, which when treated can improve balance in PD (55). Finally, the role of bradykinesia must be considered since bradykinetic-rigidity dominant PD has been identified as one of the four subtypes of PD (56, 57). Bradykinesia is a slowness of movement often observable during phasic activity such as reaching, manipulation, or stepping. Bradykinesia during slow tonic activity is more difficult to measure. Because the underlying causes of bradykinesia and rigidity are not well-understood, one can speculate whether bradykinesia falls along a spectrum from akinesia to ballismus that is inversely related to hyper- and hypotonicity. Thus, while the current study did focus on very slow movements (>60 s), the role of bradykinesia could not be completely ruled out as a contributing factor.

## Conclusion

In summary, (1) tonic postural after-effects were observed in all groups, however there were differences in amplitude and temporal properties between groups, (2) postural after-effects decayed to baseline when post-tilt surface was stable but were retained and even grew larger post-adaptation in the SR surface conditions in all groups, (3) PD participants showed less adaptation to surface changes than healthy age-matched and younger adults. Differences in size and decay of after-effects between young, old and PD groups suggest tonic neuromuscular processes play a role in how adaptable postural control is to changing surface conditions and this is affected by function of basal ganglia and associated nuclei as observed in healthy aging and neuropathology. Advancing our understanding of how posture and gait are coupled through phasic and tonic processes is a necessary step for improving rehabilitation of PD (58) and reducing fall risk in the aging population.

## Ethics Statement

Temple University Institutional Review Board Informed written consent, Protocol #12358. All participants with Parkinson's Disease were informed that this research would involve no direct benefit to them, and they could choose to not participate or stop the experiment at any time, which would have no negative impact on them.

## Author Contributions

WW conceived and designed study, collected data, performed statistical analysis, and wrote the manuscript.

### Conflict of Interest Statement

The author declares that the research was conducted in the absence of any commercial or financial relationships that could be construed as a potential conflict of interest. The reviewer CP and handling Editor declared their shared affiliation, at the time of the review.

## References

[1] Massion J. Postural control system. Curr Opin Neurobiol. (1994) 4:877–87. 10.1016/0959-4388(94)90137-6 7888772

[2] Wright WG Horak FB. Interaction of posture and conscious perception of gravitational vertical and surface horizontal. Exp Brain Res. (2007) 182:321–32. 10.1007/s00221-007-0990-4 17562029

[3] Wright WG. Tonic postural lean after-effects influenced by support surface stability and dynamics. Hum Mov Sci. (2011) 30:238–48. 10.1016/j.humov.2010.05.006 20674053

[4] Gurfinkel V Cacciatore TW Cordo P Horak F Nutt J Skoss R. Postural muscle tone in the body axis of healthy humans. J Neurophysiol. (2006) 96:2678–87. 10.1152/jn.00406.2006 16837660

[5] Magnus R Cameron prize lectures on some results of studies in the physiology of posture. Lancet (1926) 208:531–6. 10.1016/S0140-6736(01)27826-X

[6] Sherrington CS Postural activity of muscle and nerve. Brain (1915) 38:191–234. 10.1093/brain/38.3.191

[7] Takakusaki K Tomita N Yano M. Substrates for normal gait and pathophysiology of gait disturbances with respect to the basal ganglia dysfunction. J Neurol. (2008) 255 (Suppl. 4):19–29. 10.1007/s00415-008-4004-7 18821082

[8] Ivanenko Y Gurfinkel VS. Human postural control. Front Neurosci. (2018) 12:171. 10.3389/fnins.2018.00171 29615859 PMC5869197

[9] Fahn S Elton RL UPDRS Development Committee. Unified Parkinson's disease rating scale. In: Fahn S Marsden CD Calne D Goldstein M eds. Recent Developments in Parkinson's Disease. Florham Park, NJ: Macmillan Healthcare Information (1987). p. 153–63.

[10] Sheean G McGuire JR. Spastic hypertonia and movement disorders: pathophysiology, clinical presentation, and quantification. PM R (2009) 1:827–33. 10.1016/j.pmrj.2009.08.002 19769916

[11] Marciniak C. Poststroke hypertonicity: upper limb assessment and treatment. Top Stroke Rehabil. (2011) 18:179–94. 10.1310/tsr1803-179 21642056

[12] Delwaide PJ. Parkinsonian rigidity. Funct Neurol. (2001) 16:147–56. 11495420

[13] Hoehn MM Yahr MD. Parkinsonism: onset, progression and mortality. Neurology (1967) 17:427–42. 10.1212/WNL.17.5.427 6067254

[14] Bartolić A Pirtosek Z Rozman J Ribaric S. Postural stability of Parkinson's disease patients is improved by decreasing rigidity. Eur J Neurol. (2005) 12:156–9. 10.1111/j.1468-1331.2004.00942.x 15679705

[15] Wright WG Gurfinkel VS Nutt JG Horak FB Cordo PJ. Axial hypertonicity in Parkinson's disease: direct measurements of trunk and hip torque. Exp Neurol. (2007) 208:38–46. 10.1016/j.expneurol.2007.07.002 17692315 PMC2144734

[16] Franzén E Paquette C Gurfinkel VS Cordo PJ Nutt JG Horak FB. Reduced performance in balance, walking and turning tasks is associated with increased neck tone in Parkinson's disease. Exp Neurol. (2009) 219:430–8. 10.1016/j.expneurol.2009.06.013 19573528 PMC2775914

[17] Cham R Perera S Studenski SA Bohnen NI. Striatal dopamine denervation and sensory integration for balance in middle-aged and older adults. Gait Posture (2007) 26:516–25. 10.1016/j.gaitpost.2006.11.204 17196819

[18] Worapanwisit T Prabpai S Rosenberg E. Correlates of falls among community-dwelling elderly in Thailand. J Aging Res. (2018) 2018:8546085. 10.1155/2018/8546085 29992055 PMC5994309

[19] Centers for Disease Control (2018). Available online at: https://www.cdc.gov/homeandrecreationalsafety/falls/fallcost.html (Accessed May 10, 2018).

[20] Gurfinkel VS Levik Iu S Lebedev MA. [Immediate and remote postactivation effects in the human motor system]. Neirofiziologiia. (1989) 21:343–51. 2770916

[21] Aravamuthan BR Stein JF Aziz TZ. The anatomy and localization of the pedunculopontine nucleus determined using probabilistic diffusion tractography. Br J Neurosurg. (2008) 22 (Suppl. 1):S25–32. 10.1080/02688690802448251 19085350

[22] Mori S Kawahara K Sakamoto T Aoki M Tomiyama T. Setting and resetting of level of postural muscle tone in decerebrate cat by stimulation of brain stem. J Neurophysiol. (1982) 48:737–48. 10.1152/jn.1982.48.3.737 7131051

[23] Culebras A Moore JT. Magnetic resonance findings in REM sleep behavior disorder. Neurology (1989) 39:1519–23. 10.1212/WNL.39.11.1519 2812333

[24] Masdeu JC Alampur U Cavaliere R Tavoulareas G. Astasia and gait failure with damage of the pontomesencephalic locomotor region. Ann Neurol. (1994) 35:619–21. 10.1002/ana.410350517 8179307

[25] Caliandro P Insola A Scarnati E Padua L Russo G Granieri E. Effects of unilateral pedunculopontine stimulation on electromyographic activation patterns during gait in individual patients with Parkinson's disease. J Neural Transm. (2011) 118:1477–86. 10.1007/s00702-011-0705-7 21904896

[26] Thevathasan W Coyne TJ Hyam JA Kerr G Jenkinson N Aziz TZ. Pedunculopontine nucleus stimulation improves gait freezing in Parkinson disease. Neurosurgery (2011) 69:1248–53. 10.1227/NEU.0b013e31822b6f71 21725254

[27] Plaha P Gill SS. Bilateral deep brain stimulation of the pedunculopontine nucleus for Parkinson's disease. Neuroreport (2005) 16:1883–7. 10.1097/01.wnr.0000187637.20771.a0 16272872

[28] Kluzik J Horak FB Peterka RJ. Differences in preferred reference frames for postural orientation shown by after-effects of stance on an inclined surface. Exp Brain Res. (2005) 162:474–89. 10.1007/s00221-004-2124-6 15654594

[29] Walsh EG. Standing man, slow rhythmic tilting, importance of vision. Agressologie (1973) 14:79–85. 4794014

[30] Gurfinkel VS Lipshits MI Mori S Popov KE. Postural reactions to the controlled sinusoidal displacement of the supporting platform. Agressologie (1975) 17:71–76. 1008165

[31] Kluzik J Peterka RJ Horak FB. Adaptation of postural orientation to changes in surface inclination. Exp Brain Res. (2007) 178:1–17. 10.1007/s00221-006-0715-0 17039357

[32] Paquette C Paquet N Fung J. Aging affects coordination of rapid head motions with trunk and pelvis movements during standing and walking. Gait Posture (2006) 24:62–9. 10.1016/j.gaitpost.2005.07.001 16098745

[33] Schenkman M Morey M Kuchibhatla M Spinal flexibility and balance control among community-dwelling adults with and without Parkinson's disease. J Gerontol A Biol. (2000) 55A:M441–5. 10.1093/gerona/55.8.M441 10952366

[34] Akram S Frank JS Jog M. Parkinson's disease and segmental coordination during turning: I. Standing turns. Can J Neurol Sci. (2013) 40:512–9. 10.1017/S0317167100014591 23786733

[35] Vaugoyeau M Viallet F Aurenty R Assaiante C Mesure S Massion J. Axial rotation in Parkinson's disease. Journal of Neurology, Neurosurgery, and Psychiatry. (2006) 77:815–21. 16574736 10.1136/jnnp.2004.050666 PMC2117490

[36] Horak FB Frank J Nutt J. Effects of dopamine on postural control in parkinsonian subjects: scaling, set, and tone. J Neurophysiol. (1996) 75:2380–96. 10.1152/jn.1996.75.6.2380 8793751

[37] Mittelstaedt H. A new solution to the problem of the subjective vertical. Naturwissenschaften (1983) 70:272–81. 10.1007/BF00404833 6877388

[38] Roth GS Joseph JA. Cellular and molecular mechanisms of impaired dopaminergic function during aging. Ann N Y Acad Sci. (1994) 719:129–35. 10.1111/j.1749-6632.1994.tb56824.x 8010587

[39] Volkow ND Ding YS Fowler JS Wang GJ Logan J Gatley SJ Dopamine transporters decrease with age. J Nucl Med. (1996) 7:554–9. 8691238

[40] Kish SJ Shannak K Rajput A Deck JH Hornykiewicz O. Aging produces a specific pattern of striatal dopamine loss: implications for the etiology of idiopathic Parkinson's disease. J Neurochem. (1992) 58:642–8. 10.1111/j.1471-4159.1992.tb09766.x 1729408

[41] Volkow ND Gur RC Wang GJ Fowler JS Moberg PJ Ding YS. Association between decline in brain dopamine activity with age and cognitive and motor impairment in healthy individuals. Am J Psychiatry (1998) 155:344–9. 9501743 10.1176/ajp.155.3.344

[42] Vaillancourt DE Spraker MB Prodoehl J Zhou XJ Little DM. Effects of aging on the ventral and dorsal substantia nigra using diffusion tensor imaging. Neurobiol Aging (2012) 33:35–42. 10.1016/j.neurobiolaging.2010.02.006 20359780 PMC2908724

[43] Rogers MW Kennedy R Palmer S Pawar M Reising M Martinez KM. Postural preparation prior to stepping in patients with Parkinson's disease. J Neurophysiol. (2011) 106:915–24. 10.1152/jn.00005.2010 21525376

[44] Bloem BR Beckley DJ van Dijk JG Zwinderman AH Remler MP Roos RA. Influence of dopaminergic medication on automatic postural responses and balance impairment in Parkinson's disease. Mov Disord. (1996) 11:509–21. 10.1002/mds.870110506 8866492

[45] Bryant MS Rintala DH Hou JG Charness AL Fernandez AL Collins RL. Gait variability in Parkinson's disease: influence of walking speed and dopaminergic treatment. Neurol Res. (2011) 33:959–64. 10.1179/1743132811Y.0000000044 22080998 PMC5361771

[46] Foreman KB Wisted C Addison O Marcus RL Lastayo PC Dibble LE. Improved dynamic postural task performance without improvements in postural responses: the blessing and the curse of dopamine replacement. Parkinsons Dis. (2012) 2012:692150. 10.1155/2012/692150 22191075 PMC3236434

[47] Ferraye MU Debû B Fraix V Goetz L Ardouin C Yelnik J. Effects of pedunculopontine nucleus area stimulation on gait disorders in Parkinson's disease. Brain (2010) 133(Pt 1):205–14. 10.1093/brain/awp229 19773356

[48] Ferraye MU Debû B Fraix V Krack P Charbardès S Seigneuret E Subthalamic nucleus versus pedunculopontine nucleus stimulation in Parkinson disease: synergy or antagonism? J Neural Transm. (2011) 118, 1469–75. 10.1007/s00702-011-0673-y 21695419

[49] Baradaran N Tan SN Liu A Ashoori A Palmer SJ Wang ZJ Parkinson's disease rigidity: relation to brain connectivity and motor performance. Front Neurol. 2013 4:67 10.3389/fneur.2013.00067 PMC3672800 23761780

[50] Bloem BR Beckley DJ van Dijk JG. Are automatic postural responses in patients with Parkinson's disease abnormal due to their stooped posture? Exp Brain Res. (1999) 124:481–8. 10.1007/s002210050644 10090660

[51] Jacobs JV Dimitrova DM Nutt JG Horak FB. Can stooped posture explain multidirectional postural instability in patients with Parkinson's disease? Exp Brain Res. (2005) 166:78–88. 10.1007/s00221-005-2346-2 16096779 PMC1351284

[52] Jacobs JV Horak FB. Abnormal proprioceptive-motor integration contributes to hypometric postural responses of subjects with Parkinson's disease. Neuroscience (2006) 141:999–1009. 10.1016/j.neuroscience.2006.04.014 16713110

[53] Wright WG Gurfinkel VS King LA Nutt JG Cordo PJ Horak FB. Axial kinesthesia is impaired in Parkinson's disease: effects of levodopa. Exp Neurol. (2010) 225:202–9. 10.1016/j.expneurol.2010.06.016 20599976 PMC3052408

[54] Konczak J Corcos DM Horak F Poizner H Shapiro M Tuite P. Proprioception and motor control in Parkinson's disease. J Mot Behav. (2009) 41:543–52. 10.3200/35-09-002 19592360

[55] Fil-Balkan A Salci Y Keklicek H Armutlu K Aksoy S Kayihan H Sensorimotor integration training in Parkinson‘s disease. Neurosciences (2018) 23:208–15. 10.17712/nsj.2018.3.20180021 30007996 PMC8015575

[56] van Rooden SM Visser M Verbaan D Marinus J van Hilten JJ. Motor patterns in Parkinson's disease: a data-driven approach. Mov Disord. (2009) 24:1042–7. 10.1002/mds.22512 19353712

[57] Jankovic J McDermott M Carter J Gauthier S Goetz C Golbe L. Variable expression of Parkinson's disease: a base-line analysis of the DATATOP cohort. Parkinson Study Group Neurol. (1990) 40:1529–34. 10.1212/WNL.40.10.1529 2215943

[58] Mille ML Creath RA Prettyman MG Johnson Hilliard M Martinez KM Mackinnon CD. Posture and locomotion coupling: a target for rehabilitation interventions in persons with Parkinson's disease. Parkinsons Dis. (2012) 2012:754186. 10.1155/2012/754186 22295253 PMC3261491

